# *TREML2* Gene Expression and Its Missense Variant rs3747742 Associate with White Matter Hyperintensity Volume and Alzheimer’s Disease-Related Brain Atrophy in the General Population

**DOI:** 10.3390/ijms232213764

**Published:** 2022-11-09

**Authors:** Annemarie Luise Kühn, Stefan Frenzel, Alexander Teumer, Katharina Wittfeld, Linda Garvert, Antoine Weihs, Georg Homuth, Holger Prokisch, Robin Bülow, Matthias Nauck, Uwe Völker, Henry Völzke, Hans Jörgen Grabe, Sandra Van der Auwera

**Affiliations:** 1 Department of Psychiatry and Psychotherapy, University Medicine Greifswald, 17475 Greifswald, Germany; 2 Institute for Community Medicine, University Medicine Greifswald, 17475 Greifswald, Germany; 3 German Center for Neurodegenerative Diseases (DZNE), Site Rostock/Greifswald, 17489 Greifswald, Germany; 4 Interfaculty Institute for Genetics and Functional Genomics, University Medicine Greifswald, 17475 Greifswald, Germany; 5 Institute of Human Genetics, Technical University Munich, 81675 Munich, Germany; 6 Institute of Neurogenomics, Helmholtz Zentrum München-German Research Center for Environmental Health, 85764 Neuherberg, Germany; 7 Institute of Diagnostic Radiology and Neuroradiology, University Medicine Greifswald, 17475 Greifswald, Germany; 8 Institute of Clinical Chemistry and Laboratory Medicine, University Medicine Greifswald, 17475 Greifswald, Germany; 9 German Centre for Cardiovascular Research (DZHK), Partner Site Greifswald, 17475 Greifswald, Germany

**Keywords:** *TREML2*, gene expression, rs3747742, white matter hyperintensity, Alzheimer’s disease, neurodegeneration, brain atrophy, apolipoprotein E, immune system, general population

## Abstract

Although the common pathology of Alzheimer’s disease (AD) and white matter hyperintensities (WMH) is disputed, the gene *TREML2* has been implicated in both conditions: its whole-blood gene expression was associated with WMH volume and its missense variant rs3747742 with AD risk. We re-examined those associations within one comprehensive dataset of the general population, additionally searched for cross-relations and illuminated the role of the apolipoprotein E (*APOE*) ε4 status in the associations. For our linear regression and linear mixed effect models, we used 1949 participants from the Study of Health in Pomerania (Germany). AD was assessed using a continuous pre-symptomatic MRI-based score evaluating a participant’s AD-related brain atrophy. In our study, increased whole-blood *TREML2* gene expression was significantly associated with reduced WMH volume but not with the AD score. Conversely, rs3747742-C was significantly associated with a reduced AD score but not with WMH volume. The *APOE* status did not influence the associations. In sum, *TREML2* robustly associated with WMH volume and AD-related brain atrophy on different molecular levels. Our results thus underpin *TREML2*’s role in neurodegeneration, might point to its involvement in AD and WMH via different biological mechanisms, and highlight *TREML2* as a worthwhile target for disentangling the two pathologies.

## 1. Introduction

Alzheimer’s disease (AD) is a common neurodegenerative disorder affecting over 55 million people worldwide in 2021—a number which could rise up to 78 million by 2030 [1]. Brain changes associated with AD are the accumulation of amyloid beta (Aβ) plaques outside neurons and phosphorylated tau inside neurons, the degeneration of nerve cells, inflammation and atrophy [2]. Early symptoms are often impaired memory, language and thinking which may also lead to mood, behaviour and personality changes as the immutable disease progresses [2]. AD is considered to be caused by a combination of different factors with age, genetics and socioeconomic factors posing the greatest risks [2,3]. Among the genetic factors, the ε4 allele of apolipoprotein E (*APOE*) has the strongest impact on the risk of developing late-onset Alzheimer’s disease (LOAD), the most common form of AD [2]. *APOE* is a protein coding gene that is involved, among others, with tau and Aβ-related neuropathological processes and neuroinflammation [4]. An important role of the central and peripheral immune system in AD pathology is also acknowledged [5].

Increasingly recognised to be involved in the aetiology of AD are also vascular factors and cerebrovascular pathologies such as white matter hyperintensities (WMH) [6,7,8,9]. WMH are abnormalities of white matter that are frequently detected on brain magnetic resonance imaging (MRI) scans of the elderly [6,10,11]. They mostly result from chronic ischemia and cerebral small vessel disease, but chronic inflammation and glial proliferation have also been proposed as players in the yet unresolved pathogenesis [6,10,12]. The tissue damage is heterogeneous covering slight disentanglement of the matrix as well as differing degrees of myelin and axonal loss [6]. Although they are also observed in cognitively healthy younger adults [11], they have frequently been associated with an increased risk of cognitive impairment, especially regarding information processing speed, executive dysfunction, stroke, cardiovascular disease, dementia (including AD) and death [6,11,12,13,14,15,16,17,18,19,20,21]. According to population-based studies, increased age, hypertension, smoking and dyslipidemia are the strongest risk factors for WMH [6,9,12].

Due to their comorbidity, the common pathology of WMH and AD is disputed [6]. Interestingly, the gene *TREML2* (triggering receptor expressed on myeloid cells like 2) has been suggested to be implicated in both conditions. *TREML2* is a protein-coding gene located on human chromosome 6p21.1, a genomic region which has recently been associated with AD susceptibility [22]. In humans, it is expressed on lymphoid and myeloid/granuloid cells and its expression in neutrophils, macrophages and microglia is upregulated in response to inflammatory mediators [23,24,25]. Using Caucasian samples, Benitez et al. identified the *TREML2* missense variant rs3747742 as the most likely functional SNP of a GWAS meta-analysis signal associating the intergenic rs9381040 and AD risk [26,27]. The protective effect of rs3747742-C on AD was later replicated in a study of a Han Chinese population by Jiang et al. who noted that the association was present in the total sample and the subsample of *APOE* ε4 carriers, but not in the non-carriers [28]. Additionally, the missense variant has been associated with lower cerebrospinal fluid (CSF) levels of total tau (t-tau) [29] and phosphorylated tau (p-tau) [26], and the volume of the right hippocampus CA1 subfield [30]. Concerning WMH, Lin et al. found an increase in *TREML2* gene expression in whole-blood to be associated with a reduced WMH volume even when adjusting for WMH risk factors [10].

In the current study, we extend analyses on *TREML2’*s role in neurodegeneration which have previously been performed in separate studies only. Within one comprehensive data set, we use genetic, transcriptomic and MRI data of 1949 participants from the Study of Health in Pomerania (SHIP) [31] to reinvestigate the association of the expression level and missense variant of *TREML2* with (a) the WMH volume and (b) an AD score, a machine learning-generated MRI-based pre-symptomatic score measuring the resemblance of an individual’s brain atrophy with those of clinical AD cases. We thus, in particular, re-examine associations from AD case-control studies in the general population, and additionally extend previous studies by searching for cross-relations and also illuminating the role of the *APOE* ε4 status in the associations.

## 2. Results

### 2.1. Study Population

**Figure 1:**
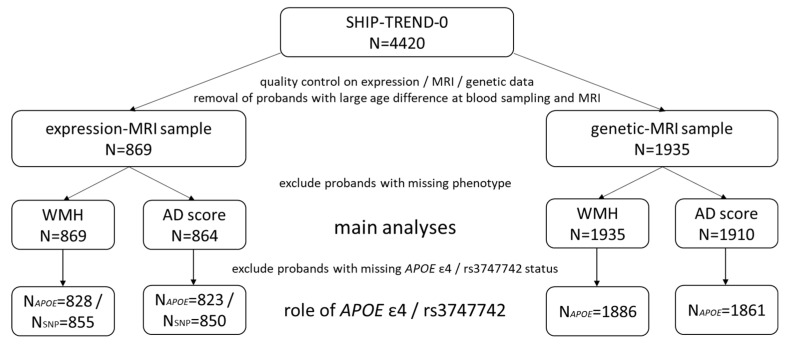
Overview of the sample sizes in the whole SHIP-TREND-0 sample and the subsamples used in this study. In total, we have used data from 1949 participants of SHIP-TREND-0. The expression-MRI and genetic-MRI samples comprise the overlap of participants with MRI data and gene expression or genetic data, respectively.

Figure 1 provides an overview of the sample sizes in the current study. A comprehensive description of the variable distribution can be found in Table 1, and a variable description in the Supplementary Materials, section Supplementary Methods. 

**Figure 2:**
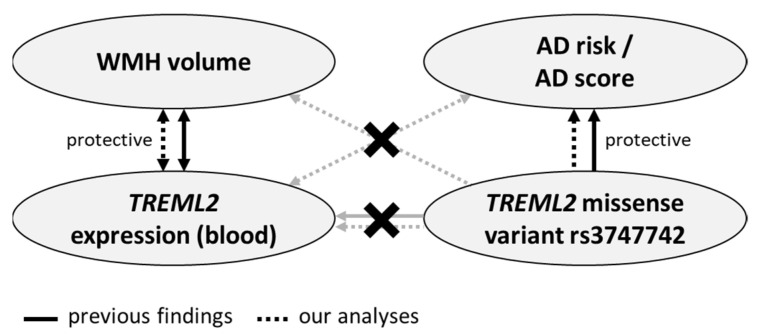
Overview of previous findings and the results of the current analyses. Crossed-out lines illustrate that no significant association has been detected. Summarized, both in our and previous studies, associations between WMH volume and whole-blood *TREML2* expression as well as AD risk and rs3747742 were detected, while rs3747742 was not found to be a whole-blood eQTL of *TREML2* in either. In addition to previous studies, we searched for cross-relations, but found none.

An overview of previous findings and our results can be found in Figure 2.

### 2.2. TREML2 Expression Associates with White Matter Hyperintensity Volume Irrespective of rs3747742 and APOE ε4 Status

As shown in Table 2, we found an increased expression of *TREML2* in whole-blood to be associated with a reduced WMH volume (β = −0.77, *p* = 0.012, N = 869). These results remained stable when additionally adjusting for either socioeconomic factors or cardiovascular factors or both. 

Details on the role of *APOE* ε4 and rs3747742 in this relationship are given in Supplementary Table S2. Briefly, when recomputing the base and the full model additionally adjusting for *APOE* ε4 status the results stayed consistent (β = −0.79, *p* = 0.012 and β = −0.82, *p* = 0.011, respectively). Also, we did not find an interaction effect of *TREML2* expression and *APOE* ε4 status onto WMH (*p* = 0.27). 

Similarly, additional adjustment of the base and full model for rs3747742 did not notably change the association between WMH volume and *TREML2* expression (β = −0.77, *p* = 0.013 and β = −0.79, *p* = 0.013, respectively). An interaction effect of *TREML2* expression and rs3747742 onto WMH volume could not be detected (*p* = 0.47).

### 2.3. TREML2 Expression Does Not Associate with the Alzheimer’s Disease Score, Neither Directly nor in Interaction with rs3747742 or APOE ε4 Status

Contrary to WMH volume, we did not find a significant direct association between the expression of *TREML2* and the AD score (*p* = 0.68, N = 864). Neither did we find interaction effects of the expression with rs3747742 (*p* = 0.70, N = 850) or *APOE* ε4 status (*p* = 0.31, N = 823) on the AD score. 

### 2.4. rs3747742 Associates with the Alzheimer’s Disease Score Irrespective of the APOE ε4 Status

As it can be seen in Table 3, there was a significant association between the *TREML2* missense variant and the AD score (β = 0.10, *p* = 0.015, N = 1910), i.e., an increased number of C alleles at rs3747742 was associated with a reduced AD resemblance of the individual’s brain. These results stayed consistent when additionally adjusting for either socioeconomic factors or cardiovascular factors or both.

When restricting the sample to the participants in TREND-Batch1, the effect direction was identical although not significant (see Table 3).

An additional adjustment of the base model and the full model for *APOE* ε4 status made only marginal changes to the association (β = 0.11, *p* = 0.010 and β = 0.11, *p* = 0.0098, respectively), and an interaction effect of rs3747742 and *APOE* ε4 onto the AD score could not be detected (*p* = 0.44). See Supplementary Table S4 for details.

### 2.5. rs3747742 Does Not Associate with White Matter Hyperintensity Volume, Neither Directly nor in Interaction with APOE ε4 Status

A direct association between rs3747742 and WMH volume could not be detected (*p* = 0.29, N = 1935). Neither did we find an interaction effect of rs3747742 and *APOE* ε4 status onto WMH volume (*p* = 0.33, N = 1886).

### 2.6. APOE ε4 Status Associates with the Alzheimer’s Disease Score but Not with White Matter Hyperintensity Volume

Assuming a positive effect direction, because the *APOE* ε4 allele is known to increase the AD risk, and thus using a one-sided significance level of 0.10 we did see a significant direct association between *APOE* ε4 and the AD score (β = 0.11, 95% CI = [−0.0049; 0.22], *p* = 0.061, N = 1861 in the base model; β = 0.11, 95% CI = [−0.0012; 0.23], *p* = 0.052, N = 1861 in the full model). However, we found no significant direct association between WMH volume and *APOE* ε4 status (*p* = 0.13, N = 1886 in the base model).

### 2.7. rs3747742 and TREML2 Expression

For the sake of completeness, we examined how rs3747742 and *TREML2* expression in whole-blood relate, but did not find a significant association (*p* = 0.63, N = 972). Also, the Genotype-Tissue Expression (GTEx) portal does not list rs3747742 as an expression quantitative trait loci (eQTL) of *TREML2* as of 2022-08-18 [32].

### 2.8. SNPs in Linkage Disequilibrium with rs3747742

We additionally analysed three SNPs, which are in linkage disequilibrium with rs3747742 (see Supplementary Table S5) and have previously been mentioned in the context of AD [27,33,34]. These are rs9357347 (within a long non-coding RNA), rs9381040 (between *TREM2* and *TREML2*) and rs6916710 (within a *TREML2* intron). Neither of them was significantly associated with WMH volume (see Supplementary Table S6). Concerning the AD score, rs9357347 and rs9381040 showed almost identical association patterns as rs3747742, and neither of the two had significant associations with the score when conditioning on rs3747742 (see Supplementary Table S7). Compared to the other two, rs6916710 was not significantly associated with the AD score, and when including it into the regression model, the association between rs3747742 and the score remained significant (see Supplementary Table S7).

## 3. Discussion

In the current study, we found an association between *TREML2* gene expression in whole-blood and WMH volume, but not our AD score measuring AD-related brain atrophy. Conversely, we found an association between the *TREML2* missense variant rs3747742 and the AD score, but not WMH volume. Neither of the two identified associations were influenced by *APOE* ε4 status. 

The identified associations are in line with previous results. Lin et al. also found increased whole-blood *TREML2* gene expression to be robustly associated with decreased WMH volume as part of a transcriptome-wide association study on 3248 participants from the Framingham Heart Study [10]. In a meta-analysis comparing 16,254 AD cases and 20,052 controls, Benitez et al. found the missense variant rs3747742-C to be associated with lower clinical AD risk in Caucasians [26]. This protective effect was later confirmed in a Han Chinese population comparing 992 LOAD patients with 1358 healthy controls [28]. We were now able to replicate this effect in a population-based dataset using a pre-symptomatic continuous MRI-based AD score that measures the resemblance of an individual’s brain atrophy patterns with those seen in AD patients [35]. 

Since the previous studies on expression~WMH and rs3747742~AD were performed in separate samples, we used the extensively phenotyped SHIP-TREND-0 sample to additionally search for cross-relations, but found no association between whole-blood *TREML2* expression and the AD score and none between rs3747742 and WMH volume. While this can have methodological reasons, it may point to the involvement of different biological mechanisms comprising either the genetic missense variant or the peripheral *TREML2* gene expression levels. This is supported by the fact that Mishra et al. [36] found no association between rs3747742 and extremes of cerebral small vessel disease (measured via WMH volume and presence of lacunes) in a genomic meta-analysis of more than 10,000 participants of European ancestry, in which SHIP-TREND-0 was included, and by the fact that rs3747742 has not been observed to be an eQTL of *TREML2* and we also found no association with *TREML2* expression in whole-blood.

In the periphery, *TREML2* is expressed in myeloid and lymphoid cells including neutrophils, macrophages and B cells [23,37,38,39]. In neutrophils, *TREML2* exhibits upregulated gene expression in response to inflammatory signals, primarily bacterial products [23,37], and specifically potentiates the response to G protein-coupled receptor agonists in mice models, leading to enhanced production of reactive oxygen species, degranulation and chemotaxis [39]. Both findings underline *TREML2’*s role in regulating the antimicrobial immune response of neutrophils. Additionally, de Freitas et al. found that activation of toll-like receptor 3, and to a lesser extent toll-like receptor 9 (two major pattern recognition receptors specialised on dsRNA and DNA from intracellular pathogens such as RNA virus), upregulates *TREML2* expression in mice on the surface of macrophages [38]. There, its protein recognises and binds to phosphatidylserine, a major “eat me” signal exposed on the surface of apoptotic cells, and hence directly and specifically mediates the ingestion of apoptotic cells by macrophages [38]. 

In the brain, studies suggest that *TREML2* is mainly expressed by microglia, which are the resident immune cells of the central nervous system [22]. While activated microglia are important in the clearance of debris, such as Aβ, chronic activation causes neurotoxicity and neurodegeneration [22]. Stimulation with lipopolysaccharide (LPS) increases TREML2 protein levels in mice microglia (in vitro) [25], as well as gene expression in mice brain (in vivo) [24] and in human primary microglia (in vitro) [24], the latter of which was also observed upon stimulation with oligomeric Aβ [24]. Additionally, *TREML2* knockdown increased microglial proliferation [24] and, in the presence of LPS, attenuated their pro-inflammatory response (assessed via levels of inflammatory cytokines, M1 and M2-type polarisation markers, and NLRP3) [24,25]. *TREML2* overexpression had opposing effects [25]. Additionally, TREML2 protein levels increase with age in the brains of AD mouse models as opposed to wild-type mice [25].

Based on the available literature and the results of this study, our hypothesis is therefore that *TREML2* increases the efficiency of immunological cleaning processes in the brain and blood before conditions turn pathological.

Concerning rs3747742, the software tools MutationTaster and PolyPhen-2 predict the amino acid change caused by the genetic variant to not affect the protein’s structure or function [40,41]. It is therefore possible that rs3747742 might not be causative, but only in linkage disequilibrium with another functional SNP. Two candidates are the intergenic rs9357347 and rs9381040, which have previously been associated with reduced AD risk and increased *TREM2* and *TREML1* gene expression levels in the temporal cortex [27,33]. Based on our dataset, we cannot favour any of the three SNPs over the other as they show almost identical association patterns with WMH and the AD score. Another candidate is rs6916710, located in a *TREML2* intron and associated with CSF t-tau and p-tau levels [34]. Here, our data suggests that rs6916710 is not the causative SNP regarding the association with the AD score.

Alternatively, as suggested also for synonymous mutations, rs3747742 might affect, to name but a few, mRNA folding and stability, translational efficiency and accuracy [42]. An influence on AD risk via AD endophenotypes is also conceivable, as the minor allele of rs3747742 has previously been associated with reduced CSF p-tau [26] and t-tau [29] levels, which are both established biomarkers of AD, as well as increased volume of the right hippocampal CA1 subfield, which might indicate a protective effect through the enhancement of brain reserves [30].

In sum, it is notable that *TREML2* is associated with neurodegeneration on different molecular levels. While the expression analysis used a quantitative measure, a missense variant might be related to protein quality. Also, despite their relatedness [7,9], WMH is a single volumetric measure reflecting vascular damage, whereas the AD score is based on AD-specific volumetric alterations of grey matter, white matter, and the ventricular system. This diversity of exposures and endpoints adds credence to *TREML2’*s role in neurodegeneration.

Our study has some limitations. Firstly, our expression data stems from whole-blood and it is unknown how *TREML2* expression in blood (directly or indirectly) affects the brain and how it relates to expression and protein levels in the brain, especially in microglia. Albeit, evidence is emerging that immunological contributions to AD pathology are not restricted to the central nervous system, but comprise the periphery as well [5,43]. Secondly, our samples in the expression and the genetic analyses are not identical as the former one is largely a subsample of the latter one. To rule out effects due to sample differences, we recalculated the genetic model on the smaller less-powered subsample and found that the effect direction was identical although not significant. Lastly, we could not independently replicate our results due to a lack of replication cohorts.

Using data from the extensively phenotyped population-based Study of Health in Pomerania we found associations of *TREML2* with WMH volume and AD-related brain atrophy on different molecular levels. Our results thus (1) underpin *TREML2’*s role in neurodegeneration, (2) might point to its involvement in AD and WMH via different biological mechanisms, and (3) highlight *TREML2* as a worthwhile target gene in the endeavour to disentangle the two pathologies. Further studies are required that analyse the interaction between *TREML2*, AD endophenotypes such as CSF p-tau and t-tau levels, pro-inflammatory markers, and neurodegeneration.

## 4. Materials and Methods

### 4.1. Study Population

The Study of Health in Pomerania (SHIP) is a population-based project with the aim to investigate disease incidences in the northeast of Germany and to analyse the relationship between risk factors, subclinical disorders and disease outcomes [31]. The current analysis is based on the SHIP-TREND-0 sample (recruitment between 2008 and 2012; aged 20 to 84 years) comprising 4420 individuals randomly drawn from the adult population of Western Pomerania, Germany [31].

### 4.2. MRI Measurements, White Matter Hyperintensity Volume and Alzheimer’s Disease Score

Whole-body MRI has been offered to all, except upon contraindication, and performed on 2159 participants from SHIP-TREND-0 using a 1.5-tesla magnetic resonance imager (Magnetom Avanto, Siemens Medical Systems, Erlangen, Germany) [44]. The examinations were executed by two trained technicians in a standardised way [44] with the following parameters: orientation = axial plane, repetition time = 5000 ms, echo time = 325 ms, slice thickness = 3 mm and resolution 0.9 mm × 0.9 mm for the T2-weighted fluid-attenuated inversion recovery (FLAIR); and axial plane, repetition time = 1900 ms, echo time = 3.4 ms, flip angle = 15° and original resolution of 1.0 × 1.0 × 1.0 mm³ for the T1-weighted magnetization prepared rapid acquisition gradient-echo (MPRAGE) sequence [45]. For more details, see Hegenscheid et al. [44] and Hosten et al. [45]. Individuals with medical conditions such as history of cerebral tumor, stroke, Parkinson’s disease, multiple sclerosis, epilepsy, hydrocephalus, enlarged ventricles or pathologic lesions, as well as individuals with poor quality of MRI scans were excluded from the analyses.

White matter hyperintensities were segmented with the lesion growth algorithm [46] as implemented in the LST toolbox version 3.0.0 for SPM using both the T1-weighted and the FLAIR MRI sequences. We set the initial threshold kappa to 0.25 and used a threshold of 0.5 to generate binary lesion maps based on the obtained probability maps to be able to extract the total lesion volume. In order to reduce the skewness of the distribution, WMH volumes (mm^3^) were log transformed, i.e., $\log_{e}\left(WMH+1\right)$.

SHIP-TREND-0 is a population-based sample with low occurrence of AD—and zero occurrence in the subsamples used for this study. It is generally recognised, however, that the brain patterns associated with the disease emerge decades before its actual onset [7,47]. We have thus used a continuous AD score, which measures the similarity of an individual’s brain atrophy pattern with those seen in clinical cases of AD [35]. This allows us to assess AD-related neurodegeneration before diagnosis or even the onset of symptoms. For a comprehensive description of the method, see Frenzel et al. [35]. Briefly, L2-penalized (ridge) logistic regression was used to train a binary classifier on MRI scans from the Alzheimer’s Disease Neuroimaging Initiative (ADNI-1 screening). The classifier optimally separates individuals with AD from cognitively normal ones using 169 brain regions of grey matter, white matter and the ventricular system [35]. The AD score is then defined as the linear predictors of the logistic model, i.e., $\log\left(\frac{p}{1-p}\right)$ with p denoting the probability of having AD [35]. Validation of the score was performed within ADNI-1 and in an independent patient sample from the Open Access Series of Imaging Studies (OASIS-1) [35]. 

### 4.3. Genetic Measurements

SHIP-TREND-0 consists of two batches. TREND-Batch1 comprises the first 1001 participants, who fasted for at least 10 h prior to blood sampling and had serum fasting glucose levels ≤ 8 mmol/L [48]. TREND-Batch2 are the remaining participants. Genotyping of the two batches was done separately [31] with N = 986 participants of TREND-Batch1 being successfully genotyped using the Illumina Human Omni 2.5 array (Illumina, San Diego, CA, USA), and N = 3133 of TREND-Batch2 using the Illumina GSA BeadChip array (Illumina, San Diego, CA, USA). Both times, the manufacturer’s recommendations were followed. After removal of single nucleotide polymorphisms (SNPs) with a Hardy-Weinberg equilibrium *p* value < 0.0001, a call rate < 0.95, and a minor allele frequency (MAF) = 0 in TREND-Batch1, and minor allele count < 10 or MAF < 1% in TREND-Batch2, imputation of missing SNPs was performed using the Haplotype Reference Consortium (v1.1, build 37) reference panel and the Eagle and minimac3 software [49,50] implemented in the Michigan Imputation Server for pre-phasing and imputation, respectively [51]. Genetic principal components were computed on the combined sample.

The missense variant rs3747742 was genotyped in both TREND-Batch1 and TREND-Batch2. The frequencies of the minor C allele in both batches are MAF = 0.33 and MAF = 0.32, respectively. The major and minor alleles are T and C, respectively.

The *APOE* ε4 status was derived from the SNPs rs429358 and rs7412 according to custom [52] (see Supplementary Table S1). rs429358 was imputed in both batches (MAF = 0.14 and imputation quality = 0.99 in both), while rs7412 was genotyped in both (MAF = 0.09 in both). 

### 4.4. Whole-Blood Transcriptome Measurements

Gene expression was only assessed on TREND-Batch1. A detailed description of blood sample collection and RNA preparation can be found elsewhere [53]. Briefly, whole-blood samples were collected from the participants after overnight fasting. Subsequent to probe preparation, the RNA was hybridised with the Illumina Human HT-12 v3 Expression BeadChip arrays and scanned with the Illumina Bead Array Reader (Illumina, San Diego, CA, USA). Reading the generated raw data, imputation of missing values and sample quality control were done using GenomeStudio V 2010 (Illumina, San Diego, CA, USA). Subsequently, the raw gene expression intensity data were normalized through quantile normalization and log2-transformation in R version 2.14.2 (The R Foundation for Statistical Computing, Vienna, Austria) [54] using the lumi 2.8.0 package [55]. Due to the used array technology and the subsequent transformations, the results cannot be interpreted quantitatively.

### 4.5. Statistical Analyses

All statistical analyses were performed in R version 4.1.1 (The R Foundation for Statistical Computing, Vienna, Austria) [54]. We used linear mixed effect models or linear regression models where applicable. If not stated otherwise the significance level is 0.05.

The base models analyse the relation between the two MRI-based phenotypes (outcome) and whole-blood *TREML2* expression, genetic measures or their interaction (exposure), respectively. All base models were adjusted for age (years), sex, and age × sex. Since age at blood sampling and age at MRI differed by up to four years within participants, we used age at blood sampling throughout the analyses while excluding participants with an age difference of more than two years (N = 1 and N = 3 for the expression-MRI and genetic-MRI sample, respectively). Also, to account for non-linear dependencies, age was modelled as restricted cubic splines in all analyses. If models contained gene expression data, they were also adjusted for white blood cells (wbc, Gpt/L), red blood cells (rbc, Tpt/L), platelets (plt, Gpt/L), neutrophils (%), monocytes (%), basophils (%), eosinophils (%), RNA integrity number (RIN), RNA amplification batch, and sample storage time (time between blood donation and RNA isolation, days), thus reducing the variability of the outcome variable that is attributable to technical parameters and blood cell composition, the latter of which is particularly relevant as our gene expression data stems from whole-blood. Amplification batch was included as a random effect. If models contained the WMH volume or the AD score, they were also adjusted for total intracranial volume (ICV, cm^3^). If they contained genetic data (SNP or *APOE* ε4 status), they were adjusted for the genetic batch and the first three genetic principal components.

To test the reliability of the results, we used three extended models. The socioeconomic model is the base model with additional adjustment for education, income, alcohol intake and partner status. The cardiovascular model is the base model with an additional adjustment for body mass index (BMI), smoking status, hypertension, serum total/hdl cholesterol ratio and triglycerides. The full model is the base model with additional adjustment for all socioeconomic and cardiovascular factors. See the Supplementary Methods for a description of these variables. The relation between whole-blood *TREML2* expression (outcome) and rs3747742 (exposure) was also analysed.

Participants with missing phenotype or missing rs3747742 or *APOE* ε4 status were excluded in the respective analysis. Missing data in covariates was imputed with the R package missForest using the whole SHIP-TREND-0 sample and all covariates except RIN, RNA amplification batch and sample storage time [56]. The variable with the highest missingness rate was income (3.8% missing in the expression-MRI and genetic-MRI sample). 94.7% and 95.0% of participants in the expression-MRI and genetic-MRI sample had a complete set of covariates.

## Figures and Tables

**Table 1:** Sample description of the study population. The columns expression-MRI sample and genetic-MRI sample refer to the current study. Markedly, restricting the SHIP-TREND-0 sample to the two subsamples did not introduce a selection bias regarding the variables relevant for our study. The variables marked with * were only measured on a subset of SHIP-TREND-0. Numerical variables are listed as mean (standard deviation), categorical variables as number (percentage). For the three main variables we additionally provide the first quartile, median and third quartile in square brackets. #NA is the number of missing values.

	SHIP-TREND-0 (N = 4420)		Expression-MRI Sample (N = 869)		Genetic-MRI Sample (N = 1935)	
	Mean (sd)/N (%)	#NA	Mean (sd)/N (%)	#NA	Mean (sd)/N (%)	#NA
**Main variables:**						
log-transformed WMH volume *	3.9 (2.8) [0, 4.3, 5.9]	2275	3.5 (2.7) [0, 4.0, 5.4]	0	3.8 (2.8) [0, 4.2, 5.8]	0
AD score *	−4.3 (1.4) [−5.3, −4.4, −3.5]	2345	−4.5 (1.3) [−5.3, −4.5, −3.8]	5	−4.3 (1.4) [−5.3, −4.4, −3.5]	25
*TREML2* gene expression *	-----	-----	7.4 (0.3) [7.2, 7.4, 7.6]	0	-----	-----
rs3747742 status (CC, CT, TT) *	413 (10%), 1805 (44%), 1902 (46%)	300	82 (10%), 387 (45%), 386 (45%)	14	192 (10%), 846 (44%), 897 (46%)	0
*APOE ε4* status (number of ε4 alleles 0, 1, 2) *	3052 (76%), 882 (22%), 87 (2%)	399	628 (76%), 181 (22%), 19 (2%)	41	1429 (76%), 414 (22%), 43 (2%)	49
**Covariates:**						
age at blood sampling (years)	52.0 (15.5)	0	50.1 (13.6)	0	50.8 (13.9)	0
sex (women)	2275 (51%)	0	484 (56%)	0	1002 (52%)	0
white blood cell count (Gpt/L)	6.2 (2.6)	15	5.7 (1.5)	1	5.9 (2.1)	2
red blood cell count (Tpt/L)	4.6 (0.4)	15	4.6 (0.4)	1	4.7 (0.4)	2
platelet count (Gpt/L)	228.1 (54.6)	15	225.9 (50.0)	1	226.5 (52.0)	2
neutrophils (%)	58.3 (8.6)	31	57.6 (8.2)	2	58.0 (8.4)	13
monocytes (%)	8.8 (2.5)	31	9.0 (2.3)	2	8.9 (2.4)	13
basophils (%)	0.5 (0.3)	203	0.5 (0.3)	3	0.48 (0.3)	44
eosinophils (%)	2.7 (1.8)	201	2.6 (1.8)	2	2.7 (1.9)	43
RNA integrity number	-----	-----	8.6 (0.5)	0	-----	-----
storage time (days)	-----	-----	216.4 (155.0)	0	-----	-----
intracranial volume (cm^3^) *	1586.8 (159.2)	2270	1576.3 (156.7)	0	1586.6 (158.3)	0
education (years)	12.2 (2.6)	68	12.8 (2.4)	7	12.7 (2.4)	14
income (€)	1361.5 (702.3)	187	1484.9 (742.1)	33	1457.4 (729.7)	73
alcohol intake (g/d)	8.4 (13.4)	51	8.5 (13.3)	7	8.1 (12.0)	16
in partnership	3418 (78%)	13	695 (80%)	2	1550 (80%)	4
body mass index (kg/m^2^)	28.1 (5.2)	7	27.1 (4.3)	0	27.5 (4.4)	0
smoking status (never, ex, current)	1605 (36%), 1610 (37%), 1183 (27%)	22	364 (42%), 318 (37%), 185 (21%)	2	762 (39%), 703 (36%), 466 (24%)	4
hypertension	2123 (48%)	17	325 (37%)	2	825 (43%)	6
serum total/hdl cholesterol ratio	4.0 (1.3)	7	3.9 (1.1)	0	4.0 (1.2)	0
triglycerides (mmol/L)	1.7 (1.2)	7	1.4 (0.9)	0	1.5 (1.1)	0

**Table 2:** WMH volume associates with *TREML2* expression. The base model has been adjusted for general participant data (age, sex, age × sex, ICV), blood parameters (wbc, rbc, plt, neutrophils, monocytes, basophils, eosinophils) and technical parameters (RIN, amplification batch, sample storage time). The socioeconomic model has additionally been adjusted for education, income, alcohol intake and partner status. The cardiovascular model is the base model with an additional adjustment for BMI, smoking status, hypertension, serum total/hdl cholesterol ratio and triglycerides. The full model is the base model with additional adjustment for all socioeconomic and cardiovascular factors.

WMH~*TREML2* Expression	Effect	95% CI	*p*-Value	N
base model	−0.77	−1.37; −0.17	0.012	869
sensitivity analyses:				
socioeconomic model	−0.79	−1.39; −0.19	0.0098	869
cardiovascular model	−0.76	−1.38; −0.15	0.015	869
full model	−0.79	−1.40; −0.17	0.012	869

**Table 3:** The AD score associates with the *TREML2* missense variant rs3747742. The base model has been adjusted for age, sex, age × sex, ICV, batch, and the first three genetic principal components. The socioeconomic model has additionally been adjusted for education, income, alcohol intake and partner status. The cardiovascular model is the base model with an additional adjustment for BMI, smoking status, hypertension, serum total/hdl cholesterol ratio and triglycerides. The full model is the base model with additional adjustment for all socioeconomic and cardiovascular factors.

AD Score~rs3747742	Effect	95% CI	*p*-Value	N
base model	0.10	0.020; 0.19	0.015	1910
sensitivity analyses:				
socioeconomic model	0.10	0.018; 0.19	0.017	1910
cardiovascular model	0.10	0.020; 0.19	0.015	1910
full model	0.10	0.019; 0.19	0.016	1910
restriction to TREND-Batch1	0.052	−0.069; 0.17	0.40	860

## Data Availability

The datasets analysed during the current study are legally owned by the University Medicine Greifswald, represented by the steering committee of the Research Network Community Medicine. Due to data protection reasons, the data is not publicly available as the comprehensive information and high sampling fraction within the regional population could enable the identification of probands [58]. Data can be applied for upon reasonable request at https://www.fvcm.med.uni-greifswald.de/dd_service/data_use_intro.php.

## Supplementary Materials

The supporting information can be downloaded at: https://www.mdpi.com/article/10.3390/ijms232213764/s1. Reference [57] is cited in the supplementary materials.
