# Supplementary material for: TREML2 Gene Expression and Its Missense Variant rs3747742 Associate with White Matter Hyperintensity Volume and Alzheimer’s Disease-Related Brain Atrophy in the General Population
Source: Int J Mol Sci. 2022 Nov 9;23(22):13764. doi: 10.3390/ijms232213764 (PMC9692564; doi:10.3390/ijms232213764)
Supplement: Supplementary file 1 [file ijms-23-13764-s001.zip › ijms-1997499-supplementary.pdf]

Supplement

Original manuscript title: *TREML2* gene expression and its missense variant rs3747742 associate with white matter hyperintensity volume and Alzheimer's disease-related brain atrophy in the general population

Authors: A. Luise Kühn, Stefan Frenzel, Alexander Teumer, Katharina Wittfeld, Linda Garvert, Antoine Weihs, Georg Homuth, Holger Prokisch, Robin Bülow, Matthias Nauck, Uwe Völker, Henry Völzke, Hans J. Grabe, Sandra Van der Auwera

Corresponding author: A. Luise Kühn, Sandra Van der Auwera

Supplementary methods: Variable description

alcohol intake – self reported alcohol intake during the last 30 days (ethanol in grams/day) [1]

education – years of education derived from the highest school degree and the highest professional degree; those participants without a finished degree were set to eight years as this was the minimal number that any other participant had stated (years)

hypertension – participant does not have hypertension if systolic blood pressure is below 140 and diastolic blood pressure is below 90 and he/she did not get a prescription for anti-hypertensives during the last year by a physician (according to ISH-WHO 1999); in all other cases the participant has hypertension (yes/no)

income – self reported sum of incomes in the household divided by the square root of the number of people living in the household (Euro)

partner status – currently living in a partnership (yes/no)

serum total/hdl cholesterol ratio – ratio between total serum cholesterol and HDL (high-density lipoprotein) serum cholesterol

smoking status – smoking status (never, ex, current)

Supplementary tables

All base models were adjusted for age (years), sex, age*sex. If they contained gene expression data, they were also adjusted for white blood cells (wbc, Gpt/l), red blood cells (rbc, Tpt/l), platelets (plt, Gpt/l), neutrophils (%), monocytes (%), basophils (%), eosinophils (%), RNA integrity number (RIN), RNA amplification batch, and sample storage time (time between blood donation and RNA isolation, days). If they contained the WMH volume or the AD score, they were adjusted for total intracranial volume (ICV, cm^3). If they contained genetic data, they were adjusted for the genetic batch and the first three genetic principal components.

The socioeconomic model is the base model with additional adjustment for education, income, alcohol intake and partner status. The cardiovascular model is the base model with an additional adjustment for body mass index (BMI), smoking, hypertension, serum total/hdl cholesterol ratio and triglycerides. The full model is the base model with additional adjustment for all socioeconomic and cardiovascular factors.

Supplementary Table S1. The APOE ε4 status was derived from the SNPs rs429358 and rs7412 according to custom [2].

rs429358	rs7412	description	*APOE ε4* status
CC	CC	Apo-e4/e4	2
CC	CT	Apo-e1/e4	1
CT	CC	Apo-e3/e4	1
CC	TT	Apo-e1/e1	0
CT	TT	Apo-e1/e2	0
TT	TT	Apo-e2/e2	0
TT	CT	Apo-e2/e3	0
TT	CC	Apo-e3/e3	0
CT	CT	Apo-e1/e3 or Apo-e2/e4	missing

Supplementary Table S2. WMH volume was associated with TREML2 expression (rows 1-4). This relationship was not notably influenced by the APOE ε4 status (rows 5-6) or the rs3747742 status (rows 8-9) of the participant. We also did not find an interaction effect of TREML2 expression with either APOE ε4 (row 7) or rs3747742 status (row 10) onto WMH volume.

White matter hyperintensity volume and *TREML2* expression					
		effect	95% CI	p-value	N
1	base model	-0.77	-1.37; -0.17	0.012	869
	sensitivity analyses:				
2	socioeconomic model	-0.79	-1.39; -0.19	0.0098	869
3	cardiovascular model	-0.76	-1.38; -0.15	0.015	869
4	full model	-0.79	-1.40; -0.17	0.012	869
	the role of *APOE ε4*:				
5	base model with *APOE ε4*	-0.79	-1.40; -0.18	0.012	828
6	full model with *APOE ε4*	-0.82	-1.45; -0.19	0.011	828
7	base model with *APOE ε4* – expression interaction	0.61	-0.47; 1.69	0.27	828
	the role of rs3747742:				
8	base model with rs3747742	-0.77	-1.37; -0.16	0.013	855
9	full model with rs3747742	-0.79	-1.42; -0.17	0.013	855
10	base model with rs3747742 – expression interaction	0.29	-0.49; 1.07	0.47	855

Supplementary Table S3. WMH volume was not significantly associated with either APOE ε4 (row 1) or rs3747742 (row 2) status.

White matter hyperintensity volume and genetics					
		effect	95% CI	p-value	N
1	base model WMH and *APOE ε4*	0.16	-0.046; 0.36	0.13	1886
2	base model WMH and rs3747742	-0.082	-0.23; 0.070	0.29	1935

Supplementary Table S4. The AD score was significantly associated with the missense variant rs3747742 (rows 1-4). This relationship was not notably influenced by the APOE ε4 status of the participant (rows 5-6). Neither did we find an interaction effect of APOE ε4 and rs3747742 onto the AD score (row 7).

**AD score and *TREML2* missense variant rs3747742**					
		**effect**	**95% CI**	**p-value**	**N**
1	base model	0.10	0.020; 0.19	0.015	1910
	sensitivity analyses:				
2	socioeconomic model	0.10	0.018; 0.19	0.017	1910
3	cardiovascular model	0.10	0.020; 0.19	0.015	1910
4	full model	0.10	0.019; 0.19	0.016	1910
	the role of *APOE ε4*:				
5	base model with *APOE ε4*	0.11	0.026; 0.20	0.010	1861
6	full model with *APOE ε4*	0.11	0.027; 0.20	0.0098	1861
7	base model with *APOE ε4* – rs3747742 interaction	-0.067	-0.24; 0.10	0.44	1861

Supplementary Table S5. Summarised information on three SNPs which are in linkage disequilibrium with the TREML2 missense variant rs3747742. MAF – minor allele frequency. The major and minor alleles are A and C for rs9357347, C and T for rs9381040, and C and T for rs6916710.

	**rs9357347**		**rs9381040**		**rs6916710**	
	TREND-Batch1	TREND-Batch2	TREND-Batch1	TREND-Batch2	TREND-Batch1	TREND-Batch2
method	imputed	imputed	genotyped	genotyped	imputed	imputed
imputation quality	0.99	0.99	--------	--------	1.00	0.99
MAF	0.34	0.33	0.33	0.32	0.39	0.38
correlation with rs3747742	0.82	0.85	0.85	0.89	0.87	0.86

Supplementary Table S6. Neither rs3747742 nor rs9357347, rs9381040 and rs6916710, which are in linkage disequilibrium with rs3747742 are significantly associated with WMH volume in our dataset.

	**effect**	**95% CI**	**p-value**	**N**
base model WMH volume ~ rs3747742	-0.082	-0.23; 0.070	0.29	1935
base model WMH volume ~ rs9357347	-0.069	-0.22; 0.082	0.37	1935
base model WMH volume ~ rs9381040	-0.098	-0.25; 0.054	0.20	1935
base model WMH volume ~ rs6916710	-0.085	-0.23; 0.061	0.25	1935

Supplementary Table S7. The candidate SNPs rs9357347 (see 2) and rs9381040 (see 3) show almost the same association pattern with the AD score as rs3747742 (see 1). When including one of the candidate SNPs together with rs3747742 neither of them is significantly associated with the AD score anymore. The candidate SNP rs6916710 (see 4) is not significantly associated with the AD score, and when included together with rs3747742 the association between the missense variant and the AD score remains significant.

		**effect**	**95% CI**	**p-value**	N
1	base model AD score ~ rs3747742	0.10	0.020; 0.19	0.015	1910

	full model AD score ~ rs3747742	0.10	0.019; 0.19	0.016	1910
2	base model AD score ~ rs9357347	0.11	0.023; 0.19	0.013	1910
	full model AD score ~ rs9357347	0.11	0.023; 0.19	0.012	1910
	base model AD score ~ rs3747742 + rs9357347				1910
	rs3747742	0.049	-0.10; 0.20	0.53	
	rs9357347	0.067	-0.085; 0.22	0.39	
3	base model AD score ~ rs9381040	0.11	0.021; 0.19	0.015	1910
	full model AD score ~ rs9381040	0.11	0.021; 0.19	0.015	1910
	base model AD score ~ rs3747742 + rs9381040				1910
	rs3747742	0.052	-0.12; 0.22	0.55	
	rs9381040	0.060	-0.11; 0.23	0.50	
4	base model AD score ~ rs6916710	0.056	-0.025; 0.14	0.18	1910
	full model AD score ~ rs6916710	0.058	-0.023; 0.14	0.16	1910
	base model AD score ~ rs3747742 + rs6916710				1910
	rs3747742	0.22	0.047; 0.38	0.012	
	rs6916710	-0.12	-0.28; 0.042	0.15	

References

[1] S. E. Baumeister, D. Alte, C. Meyer, and U. John, ‘Riskanter Alkoholkonsum und alkoholbezogene Störungen in Vorpommern: Die Studie „Leben und Gesundheit in Vorpommern” (SHIP) und der Bundesgesundheitssurvey 1998 im Vergleich’, *Gesundheitswesen*, vol. 67, no. 01, pp. 39–47, Jan. 2005, doi: 10.1055/s-2004-813829.

[2] M. Habes *et al.*, ‘Relationship between *APOE* genotype and structural MRI measures throughout adulthood in the Study of Health in Pomerania population-based cohort’, *Am. J. Neuroradiol.*, vol. 37, no. 9, pp. 1636–1642, Sep. 2016, doi: 10.3174/ajnr.A4805.
